# IDENTIFYING PATIENTS WITH PROBABLE DELIRIUM AND DEMENTIA FROM ROUTINE HOME HEALTH CARE ASSESSMENTS

**DOI:** 10.1093/geroni/igz038.443

**Published:** 2019-11-08

**Authors:** Olga F Jarrín, Abner Nyandege, Robert J Rosati, Tami J Videon

**Affiliations:** 1 Rutgers, The State University of New Jersey, New Brunswick, New Jersey, United States; 2 Rutgers University, New Brunswick, New Jersey, United States; 3 VNA Health Group, Holmdel, New Jersey, United States

## Abstract

Timely education of patients and their family caregivers on dementia disease management and health behavior changes and referral to additional resources is an essential part of age-friendly and high quality care. Nearly one out-of-three home health care patients has been diagnosed with dementia, however, family members and front-line health care providers are frequently unaware that a diagnosis of dementia has made, and what to do. The aim of this study was to identify a small set of questions that are routinely collected during home health care assessment which can be used to rapidly identify patients with suspected dementia, who may benefit from additional screening and services. We developed the preliminary model from 100% national home health care assessment data from 2014 (4.1 million people). We validated the model with a sample of nearly 27,000 patients who received a new (start-of-care) home health assessment in 2016, from four home health agencies that share a common data warehouse. The final model consisting of five questions, performed well in national data (AUC 0.85) predicting any diagnosis of dementia contained in the Medicare Chronic Conditions warehouse or home health record. The final model performed similarly (AUC 0.87) in the validation sample, predicting a diagnosis of dementia or history of dementia medication during a 3-year window of time from clinical data warehouse. The potential applications of this model have the potential to accelerate timely identification of patients with probable dementia or delirium, patient and family caregiver education, and referral to rehabilitative and supportive services.

